# Gut microbiota: an emerging target connecting polycystic ovarian syndrome and insulin resistance

**DOI:** 10.3389/fcimb.2025.1508893

**Published:** 2025-03-11

**Authors:** Yufeng Mei, Wanzhen Li, Bingqi Wang, Zhenni Chen, Xinyi Wu, Yingrui Lin, Min Wang

**Affiliations:** Department of Laboratory Medicine, The Second Xiangya Hospital, Central South University, Changsha, Hunan, China

**Keywords:** gut microbiota, PCOS, metabolic disorders, insulin resistance, potential therapy target

## Abstract

Polycystic ovary syndrome (PCOS) is a highly heterogeneous metabolic disorder, with oligomenorrhea and hirsutism as patients’ primary complaints. Hyperinsulinemia is a crucial pathophysiological mechanism in the development of PCOS, with 50-70% of patients exhibiting insulin resistance (IR). This condition not only exacerbates ovulatory dysfunction but also leads to various adverse metabolic outcomes, such as dyslipidemia and diabetes, and increases the risk of cardiovascular events both before and after menopause. Gut microbiota is a microbial community within the host that possesses significant metabolic potential and is shaped by external environmental factors, the neuro-immune network, and metabolism. Recent studies have shown that gut microbiota dysbiosis is closely related to the development and progression of PCOS. Despite the growing recognition of the potential role of gut microbiota in the pathogenesis and treatment of PCOS, its clinical application remains in its infancy. Currently, most clinical guidelines and expert consensus still emphasize traditional therapeutic approaches, such as hormonal treatments, lifestyle modifications, and insulin sensitizers. However, accumulating evidence suggests that gut microbiota may influence the metabolic and reproductive health of PCOS patients through various mechanisms. Therefore, understanding the role of gut microbiota between PCOS and IR is essential. This review describes the changes in the gut microbiota of IR-PCOS patients, examines the potential mechanisms by which the gut microbiota contributes to IR in PCOS patients, and updates the evidence supporting the gut microbiota as a potential metabolic regulatory target in IR-PCOS. In summary, gut microbiota dysbiosis may be involved in the development and progression of IR in PCOS patients, and improving gut microbiota may offer metabolic stability benefits.

## 1 Introduction

Polycystic ovary syndrome (PCOS) is a reproductive endocrine disorder characterized by irregular menstruation and elevated androgen levels, with polycystic ovaries visible on ultrasound. It affects over 15% of women of reproductive age worldwide. Hyperandrogenemia (HA) and hyperinsulinemia (HINS) are key pathological features of PCOS, influencing each other reciprocally (Bozdag et al., 2016). Up to 70% of PCOS patients exhibit insulin resistance (IR), which is linked to increased risks of gestational diabetes, miscarriage, large-for-gestational-age infants, dyslipidemia, and diabetes (El Leithy et al., 2024). This complicates clinical treatment and significantly impacts patients’ quality of life and fertility. Thus, addressing and treating IR is crucial for improving reproductive health in PCOS patients. Understanding the pathophysiology of IR in PCOS is essential for effective clinical management.

The clinical research on PCOS has expanded to the role of the gut microbiota, reflecting a growing interest in the potential of microbial imbalances, or dysbiosis, in the pathogenesis of the syndrome (Giampaolino et al., 2021). Globally, while consensus on PCOS treatment remains lacking, major organizations such as the Androgen Excess and PCOS Society (AE-PCOS) and the European Society of Human Reproduction and Embryology (ESHRE) advocate for a multidisciplinary approach, incorporating lifestyle changes, hormonal therapies, and insulin-sensitizing agents (Azziz et al., 2006; Thessaloniki ESHRE/ASRM-Sponsored PCOS Consensus Workshop Group, 2008). Recent clinical studies have explored the effects of probiotics and prebiotics on metabolic and hormonal parameters in PCOS patients, with promising findings in reducing IR and improving reproductive health (Zhang et al., 2019; Cozzolino et al., 2020). However, the integration of emerging approaches with modern clinical guidelines is still in the early stages, and further research is needed to establish the efficacy and mechanisms of these treatments in the context of gut microbiota.

Intestinal dysbacteriosis is a significant mechanism in the development of IR and PCOS (Giampaolino et al., 2021). The gut microbiota, involved in substance synthesis and metabolism, interacts with both the body and the external environment. Its complex composition is crucial for maintaining glucose and lipid metabolism and insulin sensitivity. Evidence indicates that gut microbiota is vital for insulin vesicle maturation and secretion, influencing insulin distribution through the domain-containing protein 1 (NOD1) pathway in pancreatic β-cells (Zhang et al., 2019). Moreover, the gut microbiome can enhance glucose metabolism and improve insulin sensitivity via its metabolites, affecting the neuro-immune-endocrine network (Torres-Fuentes et al., 2017). The gut microbiota also impacts female reproductive endocrinology, including menstrual cycle hormone levels, follicle development, fertilization, embryo implantation, and menopause (Qi et al., 2021). This review examines the relationship between gut microbiota and PCOS, focusing on how gut microbiota affects IR development in PCOS patients and exploring its potential as a therapeutic target. In summary, the review highlights the role of gut microbiota in the interplay between IR and PCOS, providing new insights for future interventions aimed at regulating gut microbiota to address glucose metabolism abnormalities and improve clinical outcomes in PCOS patients.

## 2 The association between IR and PCOS

### 2.1 HINS and HA

**Figure 1:**
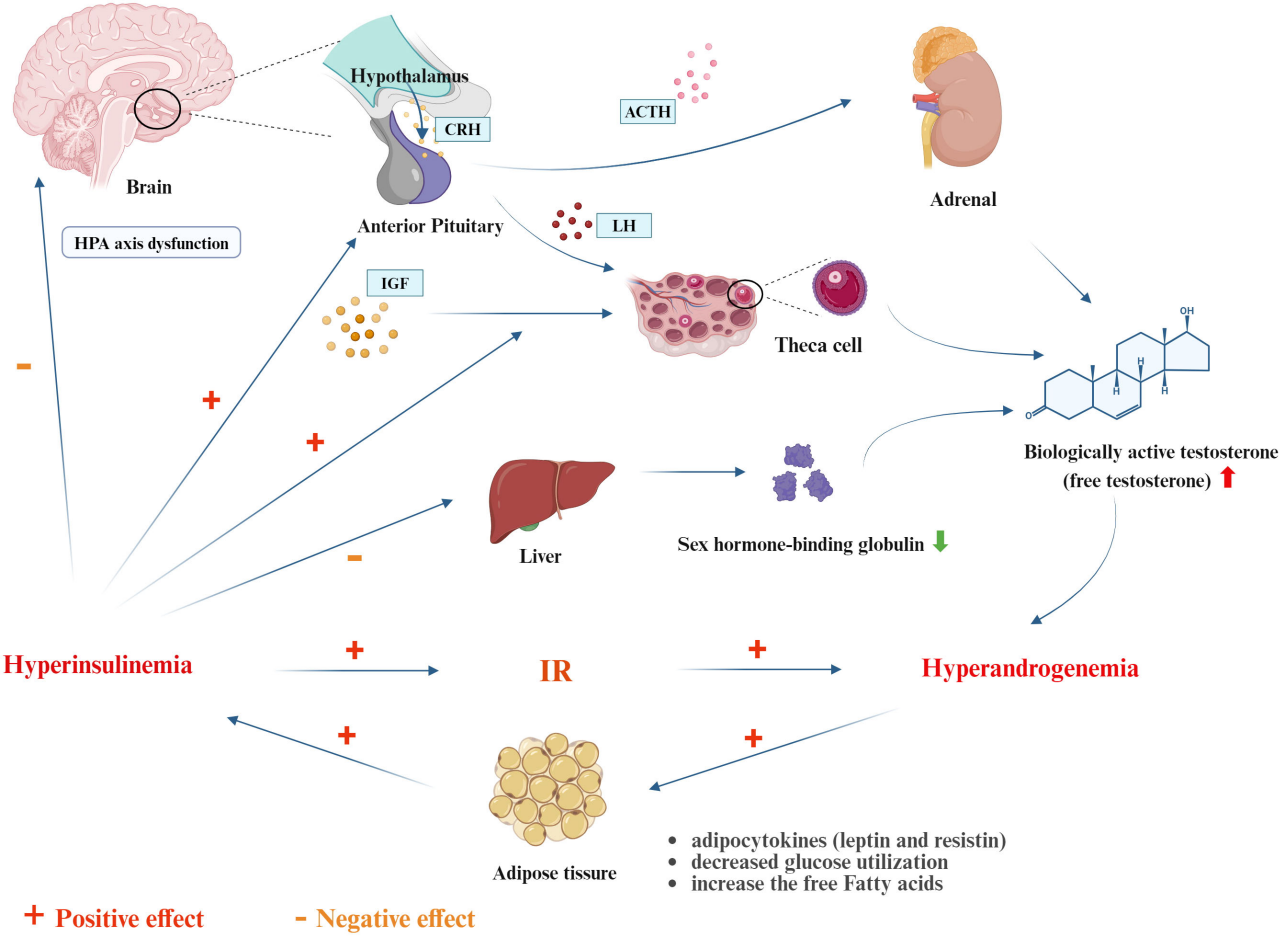
The interplay of IR between hyperinsulinemia and hyperandrogenmia (created with Biorender.com). CRH, Corticotropin-Releasing Hormone; ACTH, Adrenocorticotropic Hormone; LH, Luteinizing Hormone; IGF, Insulin-Like Growth Factor.

In patients with PCOS, the efficiency of glucose uptake and utilization decreases. The body compensates by secreting excessive insulin, leading to hyperinsulinemia (HINS) to maintain blood glucose stability. Hyperandrogenism (HA) mainly originates from the theca cells of the ovaries. Elevated levels of luteinizing hormone (LH) in PCOS patients can stimulate the ovaries to produce more testosterone, inhibit ovulation, and cause ovarian cyst enlargement. In fact, HINS and HA are the most important pathophysiological mechanisms in the development of PCOS (Rosenfield and Ehrmann, 2016; Joham et al., 2022), and they influence and depend on each other. On one hand, IR can exacerbate HA: 1) Elevated insulin levels and insulin-like growth factor (IGF) within the ovaries synergize to stimulate theca cells to produce a large amount of androgens. 2) HINS directly stimulates the pituitary to secrete LH, leading to overexpression of luteinizing hormone-releasing hormone (LHRH) and LH. LH is the primary driver of excess androgens in PCOS patients. 3) IR induces dysfunction of the hypothalamic-pituitary-adrenal (HPA) axis, increasing the response to ACTH stimulation and leading to increased adrenal androgen production. 4) The biologically active testosterone in PCOS patients is free testosterone rather than sex hormone-binding globulin (SHBG)-bound testosterone. Excessive insulin can inhibit liver synthesis of SHBG, upregulating free testosterone levels and amplifying its biological effects, making HA manifestations such as hirsutism, acne, and alopecia more pronounced. On the other hand, HA can also promote the progression of IR: 1) HA increases the conversion rate of free fatty acids in peripheral muscle tissues, inducing IR. 2) HA reduces insulin-mediated glucose utilization in muscle tissues. Dysfunctional adipocytes secrete more adipokines (such as leptin and resistin), decreasing insulin sensitivity and worsening IR. Additionally, evidence shows that anti-IR treatment in IR-PCOS patients can improve HA once insulin levels return to normal. Since insulin promotes the secretion of androgens from theca cells, controlling IR can effectively improve PCOS (**Figure 1**).

### 2.2 The influence of IR on PCOS

#### 2.2.1 IR exacerbates metabolic disorders in PCOS individuals

IR is a risk factor for increased adverse metabolic events such as non-alcoholic fatty liver disease (NAFLD), type 2 diabetes, obesity, atherosclerosis, and hypertension, involving imbalances in glucose, lipid, and amino acid metabolism networks (El Leithy et al., 2024). Approximately 70% of PCOS patients have hyperlipidemia, a common type of metabolic disorder in PCOS. Research shows that compared to non-IR-PCOS patients, those with IR-PCOS exhibit more pronounced lipid disorders, with higher levels of cholesterol, triglycerides, and LDL-C, and lower levels of HDL-C. Additionally, the level of apolipoprotein ApoC3, an indicator of lipid metabolism, is positively correlated with the degree of IR, indicating that IR can exacerbate lipid metabolism disorders in PCOS patients (Li et al., 2020). Moreover, a prolonged state of IR affects hepatic lipid metabolism, leading to decreased hormone-sensitive lipase activity and increased levels of free fatty acids in the blood. This inhibits the function of insulin-mediated glucose transporter 4 (GLUT4) and disrupts the downstream phosphoinositide 3-kinase (PI3K)-protein kinase B (Akt) signaling pathway, resulting in glucose metabolism imbalance, which further promotes the development of obesity and diabetes (Zhang et al., 2020). Peripheral muscle tissue IR can also accelerate lipolysis, with free fatty acids directly entering the liver through the portal vein, causing ectopic fat deposition in hepatocytes, impairing liver function, and inducing NAFLD (Song and Choi, 2022). IR can also affect endothelial cell integrity, accelerating the uptake of LDL-C by macrophages to form foam cells, which is a contributing factor to atherosclerosis and hypertension in PCOS patients (Toth, 2014; Hurliman et al., 2015).

#### 2.2.2 IR in reproductive dysfunction and adverse pregnancy outcomes

The primary manifestation of follicular development disorders in PCOS patients is the recruitment of an excessive number of follicles, obstruction in follicle selection and dominance, follicular development arrest, and anovulation (Bozdag et al., 2016). In contrast, the normal development of follicles involves a series of energy-consuming processes such as recruitment, selection, dominance, and ovulation. IR can disrupt energy metabolism, leading to enhanced glycolysis and inhibition of the tricarboxylic acid (TCA) cycle (Zhao et al., 2012). In the ovaries, the proliferation of granulosa cells, maturation of oocytes, and ovulation depend on the production of adenosine triphosphate (ATP) by the cells. During IR, the oocytes’ glucose metabolism and uptake are diminished (Arya et al., 2012), the glucose consumption rate in granulosa cells decreases, and the translocation of GLUT4 to granulosa cells is hindered. Consequently, lipid metabolism is enhanced to compensatorily meet the energy demands (Maruthini et al., 2014). This leads to impaired oocyte development and poor follicle quality, affecting the patient’s response to ovulation induction drugs and the outcomes of *in vitro* fertilization (IVF) (Qi et al., 2021). Moreover, these metabolic disturbances are significant factors contributing to adverse pregnancy outcomes in PCOS patients, such as gestational diabetes, preeclampsia, placental insufficiency, early miscarriage, and macrosomia. Additionally, they increase the risk of obesity and diabetes in the offspring later in life.

## 3 Gut microbiota dysbiosis and PCOS

The gut microbiota is a vast and complex ecosystem within the human gastrointestinal tract, evolved through coevolution with the host. It is estimated that the human gut harbors 100 trillion bacteria, comprising over 400 species, accounting for 78% of the total human microbiota. Due to the interactions between gut microorganisms and the human body, the gut microbiota is also referred to as the “second brain” (Lozupone et al., 2012). Under physiological conditions, the colon harbors the most diverse gut microbiota (10^^10^~10^^11^ CFU/ml), predominantly consisting of *Bacteroides*, *Firmicutes*, *Clostridia*, and *Ruminococcaceae* (Clarke et al., 2019). After birth, the human gut microbiota mainly consists of *Firmicutes*, *Actinobacteria*, *Bacteroidetes*, and *Proteobacteria*, with little fluctuation in composition (Lozupone et al., 2012; Vaiserman et al., 2017). The human gut microbiome can influence the host through microbial synthesis or metabolic degradation activities or through direct interactions between the host and microorganisms. Therefore, maintaining the balance of the gut microbiota is crucial for the proper functioning of the gut, preserving the integrity of the intestinal mucosal barrier, maintaining immune-endocrine system stability, and managing and guiding disease.

Gut microbiota dysbiosis typically refers to changes in the dynamics and function of the microbial community structure. Recent studies have shown that patients with PCOS exhibit gut microbiota imbalances. Given the individual specificity of the gut microbiota and its critical role in the immune-endocrine system and metabolic activities, changes in the gut microbiota can, to some extent, be considered as indicators of the occurrence and progression of PCOS. **Table 1** summarizes the characteristics of gut microbiota changes in PCOS patients or PCOS-like animal models based on sequencing methods. **Table 1** shows that both in humans and animals, compared to healthy controls, PCOS is associated with changes in the gut bacteria, including an increase in *Prevotella* (*Bacteroidetes*), *Shigella* and *Escherichia* (*Proteobacteria*), and *Clostridium* and *Veillonella* (*Firmicutes*), along with a decrease in some potential probiotics (*Akkermansia muciniphila*, *Bifidobacterium*, and *Roseburia*). Microbial diversity is a key indicator of gut health, where α-diversity reflects the richness and evenness of the gut microbiome, and β-diversity reflects inter-individual differences in microbial composition. Torres (Torres et al., 2018) found in humans that reduced α-diversity negatively correlates with androgen levels and that hyperandrogenism is closely linked to changes in overall bacterial composition, indicated by β-diversity. Insenser (Insenser et al., 2018) revealed gender differences in gut microbiome diversity and found that in women with PCOS, decreased α- and β-diversity correlate positively with metabolic abnormalities and hormonal imbalances, characterized by increased abundance of *Catenibacterium* and *Kandleria*. Similar findings were observed in animal models. Compared to control mice, letrozole-induced PCOS mice and androgen-exposed rats showed reduced α- and β-diversity, with decreased *Bacteroidetes* and increased *Firmicutes*, the latter being associated with IR (Kelley et al., 2016; Sherman et al., 2018). Actually, previous studies have already highlighted the role of gut microbiota in IR and PCOS. Chinese researchers analyzed the gut microbiota composition of healthy individuals, non-IR-PCOS, and IR-PCOS groups (Zeng et al., 2019). They found that PCOS patients had increased *Bacteroidaceae* and decreased *Prevotellaceae*, with IR exacerbating these differences. Additionally, compared to the non-IR-PCOS group, the IR-PCOS group showed significant differences in the abundance of *Ruminococcaceae* and *Lachnospiraceae*. Metabolic pathways in the gut microbiota, including steroid hormone biosynthesis and lipopolysaccharide biosynthesis, were notably abnormal. In conclusion, PCOS is linked to gut microbiota dysbiosis, but it remains unclear whether this dysbiosis is related to IR in the pathogenesis of PCOS.

**Table 1:** Gut microbiota investigation refers to PCOS.

Investigation	Patients/PCOS-like animal models	Controls	Methods	Intestinal flora changes
Qi et al. (2019)	50 PCOS patients	50 healthy individuals	Metagenomic sequencing	*Bacteroides vulgatus*↑, *Bacteroides dorei*↑, *Bacteroides massiliensis*↑
Rodriguez Paris et al. (2022)	8 PCOS-like C57BL/6J mice	8 control mice	postnatal exposure to DHT; bacterial 16S rRNA sequencing V4 region	*Bacteroides acidifaciens*↓
Wu et al. (2022)	6 PCOS-like SD rats	6 control SD rats	Letrozole;bacterial 16S rRNA gene sequencing V3-V4 region	*Prevotell*↑, *Veillonella*↑, *Gemella*↑, and *Fusobacterium*↑
Zhou et al. (2020b)	15 obese female with PCOS	15 obese women without PCOS	fecal 16S rRNA gene sequencing V4 region	At the Phylum level: the ratio of *Firmicutes* to *Bacteroides*↓, *Fusobacteria*↑, Tenericutes↓; At the genera level: *Lachnoclostridium*↑, *Fusobacterium*↑, *Coprococcus_2*↑ and *Tyzzerella_4*↑
Guo et al. (2016)	8 PCOS-like SD rats	8 control SD rats	Letrozole;16S rRNA gene sequencing V3 region	*Lactobacillus*↓, *Ruminococcus*↓ and *Clostridium* ↓; *Prevotella* ↑
Han et al. (2021)	6 PCOS-like SD rats	6 contro SD rats	DHEA;16S rRNA gene sequencing	*Turicibacter*↓, *Anaerofustis*↓ and *Clostridium sensu stricto*↓
Li et al. (2023)	10 PCOS-like C57BL/6J mice	10 control C57BL/6J mice	DHEA;16S rRNA gene sequencing V3-V4 regions	*Clostridia_vadinBB60_group*↑, *Enterorhabdus*↑, and *Muribaculaceae*↑
Zhou et al. (2020a)	30 PCOS women with obesity (OG); 30 PCOS women without obesity (NG)	11 healthy women with obesity (OC); 30 healthy women without obesity (NC)	16S rRNA gene sequencing V3-V4 regions	OG vs OC: *Coprococcus_2*↑;*Coprococcus_3*↓, *Lactobacillus*↓ and *Prevotella_7*↓ NG vs NC: *Tenericutes* and *Synergistets*↓, *Fusobacteria*↑.
Zhang et al. (2023)	6 PCOS-like C57BL/6J mice	6 control C57BL/6J mice	DHEA;16S rRNA gene sequencing V3-V4 regions	*Alloprevotella*↑; *Akkermansia* and *Alistipes*↓
Huang et al. (2022)	6 PCOS-like C57BL/6J mice	6 normal mice	DHEA;16S rDNA sequencing V3-V4 regions	*Akkermansia*↓; gram-negative bacteria (*Desulfovibrio* and *Burkholderia*)↑
Sherman et al. (2018)	10 Wistar rats with prenatal androgen exposure	10 healthy controls	Testosterone injection; 16S rRNA gene sequencing V3-V4 regions	bacteria associated with steroid hormone synthesis, *Nocardiaceae* and *Clostridiaceae*↑; *Akkermansia, Bacteroides*, *Lactobacillus*, and *Clostridium*↓
Eyupoglu et al. (2020)	17 PCOS patients	15 age- and BMI-match women	16S rRNA gene sequencing V3-V4 regions	*Ruminococcaceae*↑
Jobira et al. (2020)	37 PCOS adolescents	21 healthy control	16S rRNA gene sequencing V3-V4 regions	*Bacteroidaceae* and *Porphyromonadaceae*↓; *Streptococcaceae*↑
Mammadova et al. (2021)	24 lean patients with PCOS	22 BMI-matched women	16S rRNA gene sequencing V3-V4 regions	*Clostridium cluster XVII* ↑; *Clostridium sensustricto* and *Roseburia* ↓
Liu et al. (2017)	33 PCOS women	15 healthy control	16S rRNA gene sequencing V3-V4 regions	*Bacteroides*, *Escherichia/Shigella* and *Streptococcus*↑; *Akkermansia* and *Ruminococcaceae*↓
Chu et al. (2020)	14 PCOS subjects	14 healthy controls	Shotgun metagenomic sequencing	*Parabacteroides merdae*, *Bacteroides fragilis*, and strains of *Escherichia* and *Shigella*↑; *Faecalibacterium prausnitzii*↓

↑, increase; ↓, decrease.

## 4 Gut microbiota dysbiosis promote IR

### 4.1 Microorganism

The development of IR is accompanied by impaired energy utilization, and the direct metabolism of carbohydrates by microbes is one of the key sources of energy for the host. Once the physiological balance between commensal bacteria and the intestinal mucus, epithelial cells, and immune cells is disrupted, the opportunistic growth of Gram-negative bacteria and other pathogens will promote gut microbiota dysbiosis, thereby facilitating the development of IR. *Akkermansia muciniphila* is one of the microbes mediating gut barrier function and IR. It is a resident bacterium in the human gut, located in the mucus layer of the intestinal lumen and mucosal epithelium, and is an oval-shaped Gram-negative anaerobe. Under physiological conditions, *Akkermansia muciniphila* feeds on mucin secreted by the intestinal mucosa. Although its abundance only accounts for 0.5-5% of the gut microbiota, its consumption of mucin and the regeneration of mucin by goblet cells of the intestinal epithelium can reach a dynamic balance. Therefore, a decrease in its abundance can be seen as an indicator of dysbiosis (Chelakkot et al., 2018; Hasani et al., 2021). Studies have shown that the outer membrane protein Amuc_1100 of *Akkermansia muciniphila* enhances tight junctions in the intestinal epithelium through the TLR2 signaling pathway, maintaining the integrity of the gut barrier and intestinal homeostasis, and alleviating metabolic endotoxemia, thereby protecting the gut from pathogen invasion (Plovier et al., 2017). The outer membrane protein Amuc_1100 of *Akkermansia* can also promote the release of anti-inflammatory cytokines such as IL-10 by activating Toll-like receptors, regulating the host’s immune response and improving inflammation in visceral adipose tissue, thereby enhancing insulin sensitivity (Ottman et al., 2017). Additionally, the effector protein P9 of *Akkermansia* can bind to the ICAM-2 receptor on enteroendocrine cells, specifically the L cells of the intestinal epithelium, promoting the secretion of GLP-1, thus improving insulin sensitivity, lowering blood sugar, and ameliorating obesity (Yoon et al., 2021). Although current changes in the gut microbiota in PCOS, like its clinical manifestations, exhibit high heterogeneity, several PCOS-based microbiota studies have revealed that a reduction in *Akkermansia muciniphila* is associated with PCOS (Liu et al., 2017; Sherman et al., 2018; Huang et al., 2022; Zhang et al., 2023). The study by Liu R (Liu et al., 2017) and colleagues indicates that the decreased abundance of *Akkermansia muciniphila* is negatively correlated with the degree of obesity, sex hormones, particularly testosterone, and gut-brain peptides in PCOS patients. Metformin, one of the most commonly used insulin sensitizers in PCOS patients, has been shown by Huang J (Huang et al., 2022) et al. to not only mitigate microbiota dysbiosis but also increase the abundance of *Akkermansia muciniphila* in the gut, reduce serum IFN-γ levels, and inhibit pyroptosis of ovarian macrophages, thereby improving PCOS. These pieces of evidence suggest that gut microbiota dysbiosis may downregulate the abundance of *Akkermansia muciniphila*, affecting gut barrier integrity, inducing immune and metabolic imbalances, and promoting IR in PCOS patients. Supplementing the abundance of *Akkermansia muciniphila* may be a promising probiotic approach to enhancing insulin sensitivity. However, in-depth research on the effects of oral *Akkermansia muciniphila* on ovulatory dysfunction and metabolic abnormalities in PCOS is still lacking.

### 4.2 Bacterial products

#### 4.2.1 Lipopolysaccharide

LPS, also known as endotoxin, is a major component of the cell wall of Gram-negative bacteria. Due to its potent pro-inflammatory capabilities, LPS can damage pancreatic β-cell structure, impair function, and induce apoptosis, leading to insufficient insulin secretion and triggering IR (Cani et al., 2007). In PCOS patients, gut microbiota dysbiosis results in increased abundance of Gram-negative bacteria such as *Bacteroides*, *Escherichia coli*, *Desulfovibrio*, and *Burkholderia* (Liu et al., 2017; Chu et al., 2020). During this time, the gut microbiota serves as a reservoir for LPS, leading to elevated LPS levels in the intestinal lumen, which can increase gut mucosal barrier permeability, induce gut leakage, and promote the translocation of gut-derived LPS into the bloodstream, causing metabolic endotoxemia. On one hand, elevated circulating LPS levels can directly interfere with insulin receptor function, leading to hyperinsulinemia and exacerbating the IR process (Saad et al., 2016). On the other hand, LPS can bind with lipopolysaccharide-binding protein (LBP), widely engaging with CD14 on T cells, B cells, macrophages, and natural killer cells, subsequently presenting LPS to the transmembrane protein TLR4. This interaction activates signaling molecules such as MyD88, IRAK, IRAK2, and TRAF6, promoting the production of inflammatory cytokines (IL-1, IL-6, and TNF-α), which in turn amplify LPS’s pro-IR capacity through positive feedback (Raetz and Whitfield, 2002; Gnauck et al., 2016; Saad et al., 2016). IL-1, a key mediator of inflammation, can disrupt the proliferation and induce the apoptosis of pancreatic β-cells (Herder et al., 2015). IL-6, a multifunctional glycoprotein factor involved in ovulation and post-ovulatory ovarian repair, is elevated in PCOS patients and can activate the Janus kinase pathway, increasing the expression of suppressor of cytokine signaling-3 (SOCS-3), which inhibits insulin signaling. This reduces GLUT-4 secretion and inhibits tyrosine phosphorylation of insulin receptor substrate (IRS)-1, thereby blocking the insulin signaling pathway and inducing IR. Elevated TNF-α levels can lead to abnormal serine phosphorylation of IRS-1, disrupting IRS protein signaling and stimulating IR in adipose tissue (Fulghesu et al., 2011; Rudnicka et al., 2021). Furthermore, studies have shown that in PCOS mice with IR, the expression of tight junction proteins Occludin and ZO-1 decreases while LPS levels increase (Guan et al., 2024). Therefore, gut microbiota dysbiosis can induce metabolic endotoxemia and amplify systemic inflammatory responses, promoting the occurrence of IR in PCOS patients.

#### 4.2.2 Trimethylamine N-oxide

**Figure 2:**
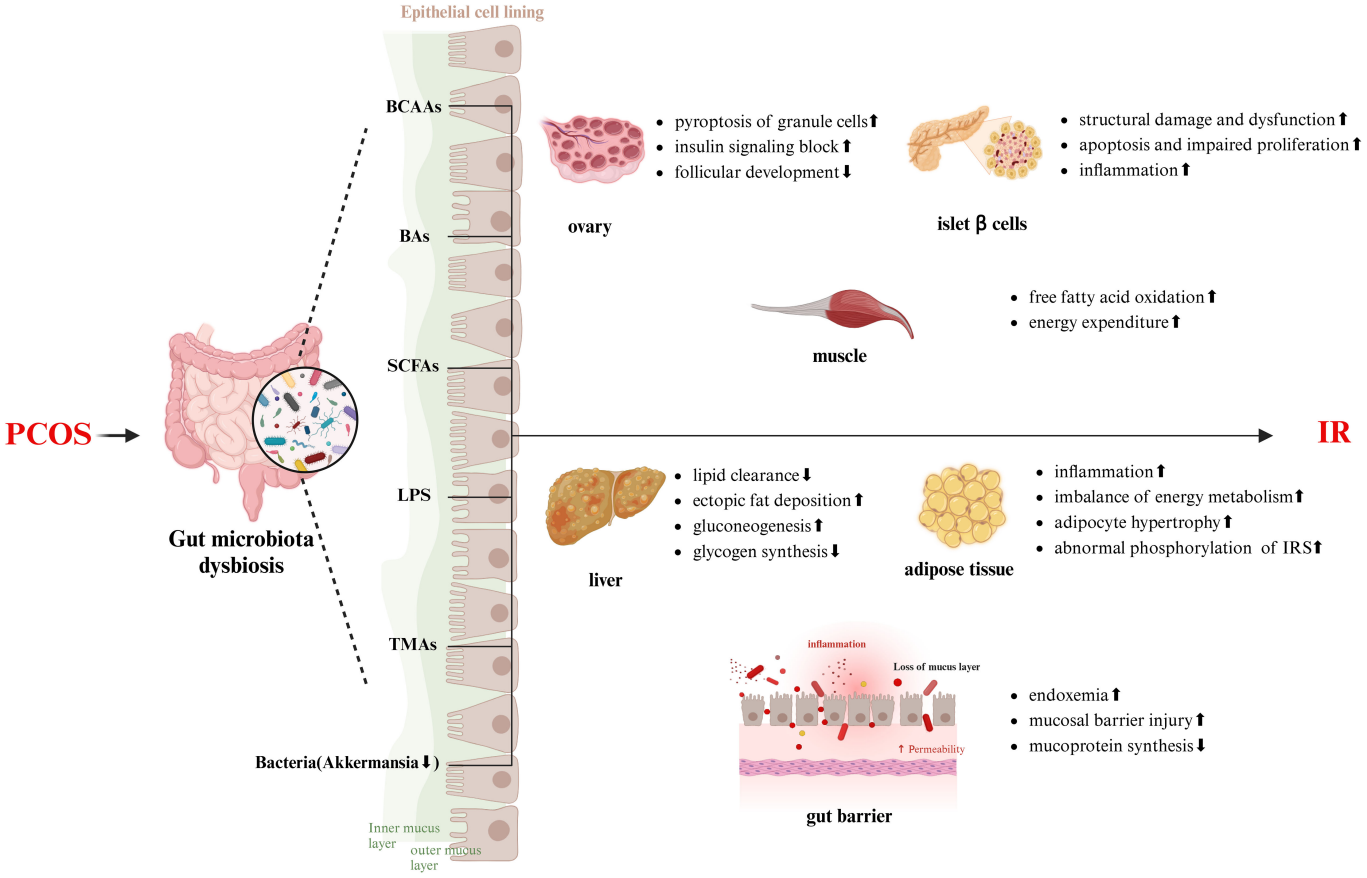
The mechanism of gut microbiota in the development of IR in PCOS patients (created with Biorender.com). IRS, insulin receptor substrate.

TMAO is a phospholipid metabolite related to gut microbiota metabolism, primarily derived from *Firmicutes*, *Proteobacteria*, and *Actinobacteria* (Falony et al., 2015). Dietary choline (such as L-carnitine and phosphatidylcholine) is converted to trimethylamine (TMA) by gut microbiota, particularly hydrogenotrophic anaerobes, *Asaccharobacter*, *Clostridium* sp*orogenes*, and *Proteobacteria*. Absorbed TMA is further converted to TMAO in the liver by flavin monooxygenase 3 (FMO3) (Zhuang et al., 2019). Serum levels of TMAO and its precursors are significantly elevated in PCOS patients compared to healthy women and correlate positively with testosterone levels, as well as long-term dysregulation of glucose and lipid metabolism in PCOS patients. In fact, elevated TMAO is directly associated with increased IR risk; dietary interventions or targeting gut microbiota can effectively reduce blood levels of TMAO and its precursors, improving fasting glucose and HOMA-IR (Heianza et al., 2019). These findings suggest that gut microbiota play a significant role in PCOS and IR via TMAO. However, the mechanisms by which TMAO contributes to PCOS and IR remain unclear and may involve the following: 1) inducing the expression of membrane proteins (CD36 and scavenger receptor A), inhibiting cholesterol reverse transport, leading to lipid clearance disorders (Geng et al., 2018); 2) triggering the release of inflammatory mediators (such as TNF-α, IL-6, and IL-18), activating the TXNIP-NLRP3 pathway and NF-κB, which is crucial for mediating inflammatory damage and apoptosis of pancreatic β-cells (Gil-Cruz et al., 2019); 3) inhibiting bile acid synthesis, reducing cholesterol clearance, promoting adipocyte proliferation, and IR (Lu et al., 2010); 4) impairing hepatic insulin signaling, increasing gluconeogenesis (**Figure 2**).

#### 4.2.3 Bile acids

BAs are essential digestive fluids in the human body, and their synthesis, metabolism, and biotransformation are closely related to gut microbiota. The gut microbiota convert primary BAs (cholic acid and chenodeoxycholic acid) into secondary BAs [deoxycholic acid (DCA), lithocholic acid (LCA), and ursodeoxycholic acid (UDCA)] through dehydroxylation, involving bacteria such as Lactobacillus, Bifidobacterium, and Listeria (Fukui, 2015). Dysbiosis can lead to decreased BA activation, inhibiting glucose metabolism and insulin sensitivity through various signaling pathways. When BA production is impaired, bacterial overgrowth in the small intestine and increased intestinal permeability can lead to the entry of toxic substances into the systemic circulation, causing endotoxemia and inducing IR. This suggests a mutual promotion of dysbiosis and BA imbalance (Saad et al., 2016). One study showed that serum levels of glycine- and taurine-conjugated primary BAs were significantly elevated in PCOS individuals, with elevated circulating conjugated primary BAs levels positively correlated with hyperandrogenemia (Zhang et al., 2019). Another study indicated significant alterations in BA metabolic profiles in the follicular fluid of PCOS patients (Yang et al., 2021), suggesting BA metabolic dysregulation in PCOS, though the mechanisms require further investigation. Qi X et al. found (Qi et al., 2019) that feces from PCOS patients could induce ovarian cysts and IR in mice and had a high capacity for bile salt deconjugation. This resulted in significantly reduced levels of conjugated secondary BAs—GDCA and TUDCA—due to an increased abundance of Bacteroides with bile salt hydrolase activity in the gut of PCOS patients. Exogenous supplementation with GDCA and TUDCA improved the PCOS phenotype in mice, possibly related to the regulation of the BAs/IL-22 signaling axis. Additionally, dysfunction of the Farnesoid X receptor (FXR) and Takeda G-protein receptor 5 (TGR5) might be involved. BAs are ligands for FXR and TGR5 in the liver and intestine, and their activation can (Lefebvre et al., 2009): (1) stimulate insulin secretion; (2) promote GLP-1 secretion from intestinal L-cells, improving hepatic glucose metabolism and IR; (3) activate glycogen synthase kinase (GSK), accelerating glycogen synthesis; (4) regulate appetite by affecting vagal afferent neurons (VANs) through the gut-brain axis; and (5) inhibit LPS-induced inflammatory responses. In PCOS patients, dysbiosis leads to an imbalance and reduced activation of BAs, disrupting glucose metabolic pathways involving FXR and TGR5, thereby promoting the development of IR.

#### 4.2.4 Branched-chain amino acid

BCAAs are essential dietary amino acids metabolized by gut bacteria, primarily including valine, leucine, and isoleucine. They are directly absorbed by the intestine without hepatic metabolism. In the human gut, the fermentation bacteria for BCAAs are *Clostridium*, *Bacillus-Lactobacillus-Streptococcus*, and *Proteobacteria* (Dai et al., 2011). Multiple studies have shown elevated levels of BCAAs in the peripheral blood and follicular fluid of PCOS patients, with BCAA concentrations significantly correlated with BMI, HOMA-IR, waist circumference, and total testosterone (Mu et al., 2023; Paczkowska et al., 2023). When classified by the presence of IR, the IR-PCOS group exhibited higher levels of leucine, valine, and glutamate compared to the non-IR-PCOS and healthy groups. High BCAA levels were also associated with lower pregnancy rates and higher miscarriage rates (Zhang et al., 2014). In animal studies, dietary BCAA supplementation in mice increased the risk of developing IR and diminished the beneficial effects of exercise on glucose and lipid metabolism (Zhang et al., 2022). In fact, the relationship between high BCAA levels and IR was described as early as 30 years ago, but only recently has a scientific understanding emerged (Eriksson and Björkman, 1993). Dysbiosis is a key factor in this relationship. PCOS patients exhibit gut microbiota dysbiosis, with higher abundances of *Streptococcus* and *Prevotella*, which are major BCAA-producing genera (Yue et al., 2019; Jobira et al., 2020). Yu Z et al. found that structural and functional changes in the microbiota drive the development of glucose intolerance in rats, raising serum BCAA levels, and suggested that BCAA-induced IR may be related to the mammalian target of rapamycin (mTOR) signaling pathway (Yu et al., 2024). Gojda J et al. further indicated that BCAAs can increase the oxidation of free fatty acids, activating the mTOR/PI3K/protein kinase B (PKB) pathway, thereby inducing IR (Gojda and Cahova, 2021). In summary, dysbiosis leading to elevated BCAA levels may contribute to IR development by affecting the mTOR pathway.

#### 4.2.5 Short-chain fatty acid

SCFAs, primarily acetate, propionate, and butyrate, are produced by the fermentation of dietary fibers such as carbohydrates and proteins by gut microbiota. Acetate is produced by Bacteroidetes, propionate by *Veillonella*, *Bacteroides*, and *Salmonella*, and butyrate by *Faecalibacterium prausnitzii*, *Anaerostipes* spp, Roseburia spp, and *Eubacterium* spp (Mann et al., 2024). Butyrate-producing bacteria play a major role in improving IR. Multiple studies have revealed that the abundance of butyrate-producing bacteria is reduced in individuals with IR, and increasing their abundance significantly alleviates inflammation and metabolic disorders in IR individuals (Bridgeman et al., 2020; Mann et al., 2024). Gut dysbiosis in PCOS patients is associated with reduced SCFA levels, which interact with G protein-coupled receptor 43 (GPCR43) and GPCR41 in enteroendocrine cells, intestinal epithelial cells, and pancreatic β-cells, increasing the secretion of glucagon-like peptide-1 (GLP-1) and peptide YY, regulating the hypothalamic-pituitary-gonadal axis, and altering luteinizing hormone and testosterone levels, thereby affecting the progression and phenotype of PCOS (Wang et al., 2021). Reduced abundances of *Akkermansia muciniphila*, *Blautia*, and *Roseburia* in PCOS patients result in decreased SCFA synthesis, leading to reduced expression of ghrelin and peptide YY (PYY). Probiotic treatment in PCOS patients increases SCFA levels and restores blood glucose homeostasis (Zhang et al., 2019). This indicates that SCFAs not only influence hormonal imbalances in PCOS but are also crucial in the development of IR-PCOS. Additionally, SCFAs activate peroxisome proliferator-activated receptor gamma (PPARγ), increase the expression of tight junction proteins in the intestinal mucosal barrier, and inhibit intestinal epithelial cell apoptosis, promoting fatty acid oxidation and intestinal mucosal immunity (Zhang et al., 2022). Therefore, gut dysbiosis leading to decreased SCFA levels contributes to the development of IR in PCOS patients.

## 5 Regulate gut microbiota as the possible therapy target for IR-PCOS

### 5.1 Probiotics and prebiotics

**Figure 3:**
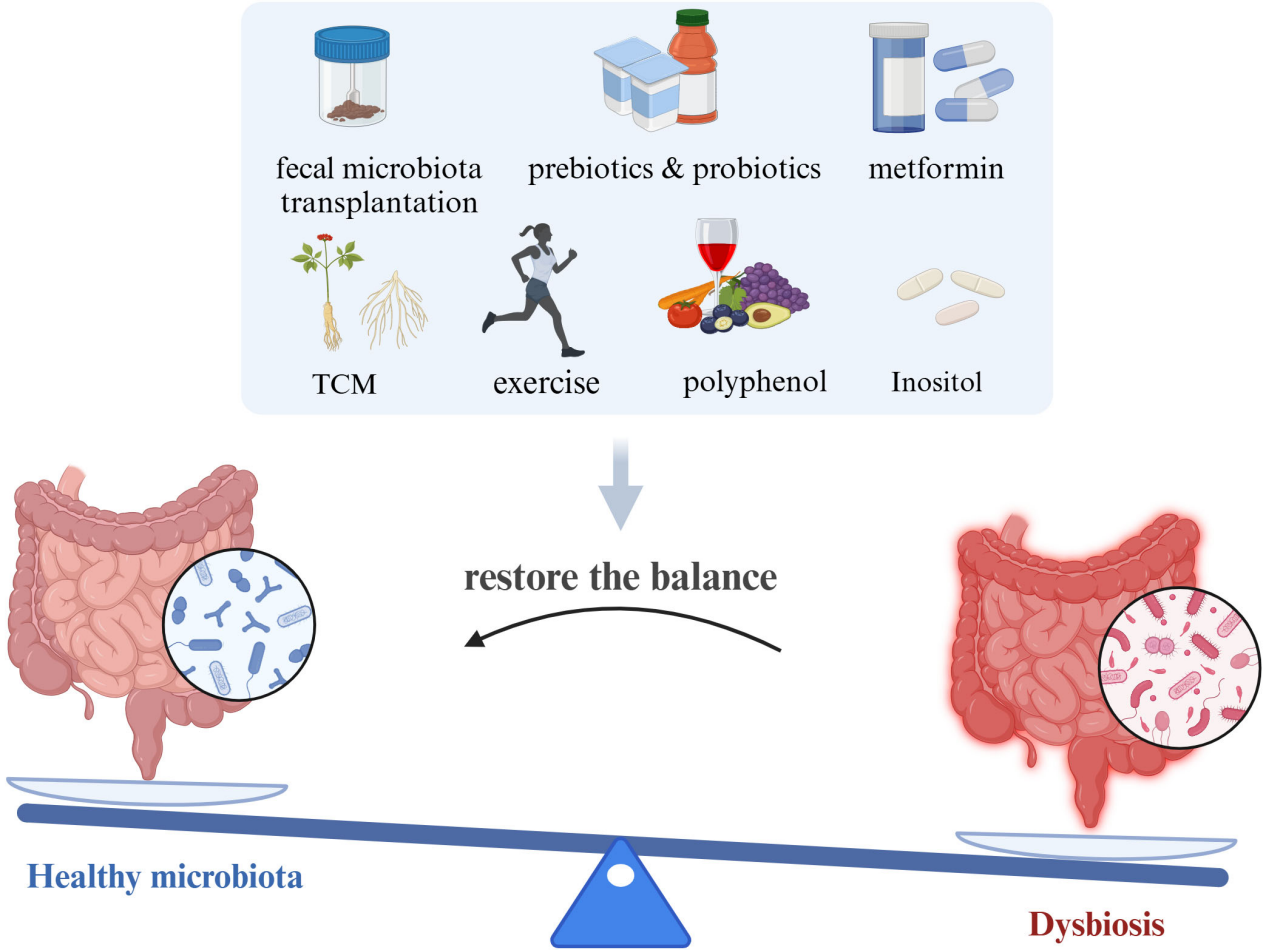
Potential methods targeted on gut microbiota for improving IR-PCOS (created with Biorender.com).

Probiotics are active microorganisms present in the human gut that confer beneficial effects by maintaining microbial balance or modulating host immunity, including *Bifidobacterium* and *Lactobacillus* species. Prebiotics are organic substances that selectively promote the metabolism and proliferation of probiotics in the gut, thereby improving the dynamics and metabolic functions of the gut microbiota. Increasing evidence suggests that probiotics and prebiotics have significant advantages in treating IR-PCOS (Li et al., 2022; Luo et al., 2023). At the human level, a decrease in *Bifidobacterium* abundance is a characteristic of dysbiosis in PCOS patients. Supplementing with *Bifidobacterium lactis V9* can promote the growth of SCFA-producing microorganisms such as *Faecalibacterium prausnitzii*, *Butyricimonas*, and *Akkermansia*, which can improve gut health by enhancing barrier function and reducing the translocation of bacterial endotoxins across the gut wall. These endotoxins can cause inflammation and IR in the gut wall (Zhang et al., 2019). Supplementing with a multi-strain probiotic formulation (including *Lactobacillus casei*, *Lactobacillus acidophilus*, *Lactobacillus paracasei*, and *Bifidobacterium bifidum*) not only lowers fasting blood glucose, HOMA-IR, and dyslipidemia but also improves systemic inflammation levels and hyperandrogenism in patients (Cozzolino et al., 2020). At the animal level, similar effects have been observed with probiotics or prebiotics. Intake of probiotics and prebiotics increased the relative abundance of *Lactobacillus*, *Bifidobacterium*, and *Akkermansia*, improving reproductive dysfunction and metabolic abnormalities in PCOS phenotype mice (Li et al., 2022). *Escherichia coli Nissle 1917* (EcN), a genetically controlled probiotic, increased the abundance of *Adlercreutzia*, *Allobaculum*, *Escherichia-Shigella*, and *Ileibacterium* in PCOS mice, improving metabolic disorders by enhancing amino sugar and nucleotide sugar metabolism pathways (Luo et al., 2023). Despite the promising benefits of probiotics and prebiotics observed in various clinical trials and animal studies for IR-PCOS, the diverse range of probiotic strains, dosages, and treatment durations lack standardization. Therefore, further research is needed to refine the use of probiotics and prebiotics for optimal therapeutic protocols (**Figure 3**).

### 5.2 Fecal microbiota transplantation

Fecal microbiota transplantation (FMT) aims to reshape the gut microbiota by transplanting fecal microbiota from healthy donors into the patient’s gut, thereby enhancing the host’s gut mucosal immunity. FMT has shown promising results in treating diseases such as inflammatory bowel disease and recurrent Clostridium difficile colitis. However, current research primarily focuses on fecal transplantation in individuals with PCOS. Both fecal samples from PCOS patients and PCOS phenotype animals have shown strong PCO and IR-inducing abilities. Qi X et al. found that mice gavaged with fecal matter from PCOS patients exhibited IR, disrupted estrous cycles, increased cystic follicle numbers, and decreased corpora lutea, a phenomenon associated with the high abundance of Bacteroides in the PCOS gut (Qi et al., 2019). At the animal level, Han Q et al. discovered that transplanting the gut microbiota from DHEA-induced PCOS phenotype rats into germ-free recipients promoted IR and reproductive hormone imbalance (Han et al., 2021). Guo Y et al. performed FMT in PCOS phenotype rats and found that the PCOS-like rats had lower levels of *Lactobacillus*, *Ruminococcus*, and *Clostridium*, while *Prevotella* was more abundant. After administration of probiotics containing *Lactobacillus* and FMT, the gut microecological structure was restored, and metabolic abnormalities and hormone imbalances were ameliorated (Han et al., 2021). Additionally, in diabetes characterized by IR, FMT significantly reversed IR and enhanced the insulin-sensitizing effects of metformin (Wu et al., 2023). Therefore, modulating gut microbiota holds promise for treating IR-PCOS. In summary, while it is widely recognized that PCOS patients exhibit gut microbiota imbalance, FMT research in PCOS is still in its early stages globally. The application of FMT in IR-PCOS faces several challenges, including the anaerobic handling and preservation of donor feces, donor-recipient incompatibility, and the uniformity and effectiveness of donor feces recolonization in recipients. These challenges necessitate careful and comprehensive evaluation from both clinical and mechanistic perspectives.

### 5.3 Polyphenol

Polyphenols, found in natural plants, are compounds with antioxidant and anti-inflammatory properties, including flavonoids and non-flavonoids. Polyphenols can reduce inflammation and stabilize glucose and lipid metabolism in IR-PCOS by promoting beneficial gut bacteria and reducing potential pathogens (Zhou et al., 2024). Gut microbiota can metabolize polyphenols through hydrolysis and reduction reactions, and the beneficial effects of polyphenols on PCOS may arise from aromatic metabolites produced by gut microbiota metabolism. In recent years, polyphenols involved in regulating IR-PCOS mainly include resveratrol, catechins, and anthocyanins, with resveratrol being the primary polyphenol affecting gut microbiota. Resveratrol is generally considered a scavenger of reactive oxygen species and free radicals, offering some protection to the ovaries, and its value in improving IR in PCOS has been extensively studied (Pasquariello et al., 2020; Mansour et al., 2021; Chen et al., 2022). Chen M et al. found that resveratrol improves ovarian ovulation disorders in PCOS patients by reducing granulosa cell apoptosis and oxidative stress levels (Chen et al., 2022). In addition to reducing oxidative stress, resveratrol can reverse pyruvate and lactate levels, upregulate key rate-limiting enzymes related to glycolysis pathways (such as LDHA, HK2, and PKM2), activate the SIRT2 signaling pathway, and improve IR-PCOS (Chen et al., 2022; Liang et al., 2023). To further explore the relationship between resveratrol and gut microbiota, Chinese researchers evaluated the effect of resveratrol on follicle development through FMT. Evidence shows that resveratrol regulates the SIRT1-FoxO1/P53 pathway to reduce follicle atresia, a process involving changes in gut microbiota. After transplanting the gut microbiota from resveratrol-treated donors, the *Firmicutes*/*Bacteroidetes* ratio significantly increased, along with the relative abundance of *Lactobacillus murinus* and *Lactobacillus salivarius* (Wang et al., 2022). Another study found that resveratrol significantly improved IR in high-fat diet mice by reducing endotoxemia, inflammation, and restoring gut barrier defects, while increasing the abundance of *Verrucomicrobia* and *Akkermansia* (Chen et al., 2020). These findings suggest that resveratrol may treat IR-PCOS by improving gut microbiota. However, due to the vast variety of polyphenols, there are still many limitations and challenges in verifying their mechanisms and efficacy. In conclusion, it is certain that FMT may be effective for PCOS, but it only remains at the level of clinical practice and its future use in humans remains to be considered.

### 5.4 Metformin

Metformin is the most commonly used insulin sensitizer in clinical practice for PCOS patients. Apart from inhibiting endogenous glucose production and improving IR, it also plays a role in alleviating hyperandrogenism in PCOS patients, facilitating ovulation restoration, and reducing long-term pregnancy and metabolic risks (Furat Rencber et al., 2018). Recent findings indicate that increasing gut microbiota diversity and abundance is one of the mechanisms of metformin’s action. Following metformin treatment, PCOS patients show significantly increased abundance of beneficial bacteria such as *Bacteroidetes*, *Proteobacteria*, *Hungatella*, *Phocaeicola*, *Anaerobutyricum*, predominantly *Clostridium*, *Fusobacterium*, and *Oxalobacter* (Gan et al., 2023). Animal experiments demonstrate that metformin reduces LH, LH/FSH, and TNF-α levels in PCOS phenotype mice, improves the number of ovarian atretic follicles and Graafian follicles, and upregulates *Akkermansia* abundance in the gut while lowering serum IFN-γ levels. This mechanism may involve reducing gut LPS endotoxemia and enhancing inhibition of IFN-γ-induced macrophage pyroptosis (Zhou et al., 2016; Furat Rencber et al., 2018; Huang et al., 2022). Importantly, gut *Akkermansia* appears to be a target of metformin’s action, and its role in improving IR is widely recognized. *In vitro* studies show that adding metformin promotes *Akkermansia* growth (Ropot et al., 2020). At the animal level, metformin-treated IR mice exhibit improved glucose profiles, increased mucin layer thickness in the intestines, and higher *Akkermansia* abundance. Oral administration of *Akkermansia* to mice results in similar improvements in glucose tolerance as metformin treatment (Shin et al., 2014). These findings suggest that metformin improves IR-PCOS by increasing the abundance of beneficial gut bacteria.

### 5.5 Exercise

Whether lean-type or obese-type PCOS, physical exercise is the most effective and economical method for treating menstrual irregularities, hormonal disturbances, and metabolic abnormalities in PCOS patients, linked to enhancing immune function, maintaining neuroendocrine balance, and reducing inflammation (Sivasankari and Usha, 2022). Studies indicate that regular physical exercise can improve menstrual irregularities and reproductive disorders in 50% of women with PCOS (Lim et al., 2019). However, there is currently a lack of evidence on whether exercise affects gut microbiota and thereby regulates IR-PCOS. During exercise, the internal environment undergoes dynamic changes, allowing beneficial bacteria to proliferate rapidly in response to the homeostatic and physiological changes induced by exercise (Mika et al., 2015). Therefore, exercise can shape the microbiota independently of diet. Compared to non-athletes, athletes show higher α-diversity of gut microbiota, and the abundance of species such as *Bifidobacterium*, *Lactobacillus*, *Prevotella*, and *Faecalibacterium* varies with different exercise intensities, indicating that exercise influences microbiota composition (O’Donovan et al., 2020; O’Brien et al., 2022). For PCOS, static exercises like yoga and meditation can improve glycemic and lipid metabolism and IR status in adolescent PCOS females, increase melatonin secretion, and effectively regulate endocrine and metabolic disorders (Harinath et al., 2004; Nidhi et al., 2012). Imbalances in gut microbiota can affect neuroendocrine networks, including the hypothalamic-pituitary-ovarian axis, thereby influencing melatonin secretion (Iesanu et al., 2022). In conclusion, regardless of the type of exercise, physical exercise may alter gut microbiota composition and affect IR-PCOS.

### 5.6 Traditional Chinese medicine

TCM in the treatment of PCOS, particularly in the context of gut microbiota modulation, has gained increasing attention. TCM, which includes herbal medicine, acupuncture, and dietary therapy, has been practiced for centuries in China and is recognized for its potential in addressing the complex pathophysiology of PCOS. Recent studies suggest that certain TCM formulations may exert therapeutic effects by altering gut microbiota composition. For instance, Wang et al. demonstrated that a TCM herbal formula can alleviate metabolic abnormalities in PCOS rats by targeting the intestinal LPS/TLR4 pathway (Wang et al., 2022). Moreover, individual herbs such as Astragalus have been linked to changes in gut microbiota, potentially reducing IR and oxidative stress in PCOS patients (Li et al., 2024). Acupuncture, another key component of TCM, has shown promise in early studies by Fan et al., which suggest that it may improve PCOS symptoms through gut microbiota modulation, though the mechanisms remain unclear (Fan et al., 2021). Dietary therapy, a fundamental aspect of TCM, emphasizes the consumption of foods that promote health and balance. Preliminary evidence indicates that fiber-rich diets recommended in TCM may support beneficial gut bacteria growth and alleviate PCOS symptoms (Leung et al., 2022). Although research on TCM and its effects on the gut microbiota in PCOS is still in its early stages, these findings underscore the potential of integrating TCM with modern treatment strategies for a more comprehensive approach to PCOS management.

### 5.7 Inositol

Inositol, a naturally occurring cyclitol, exists predominantly in its active forms, myo-inositol (MI) and D-chiro-inositol (DCI), within the human body, playing a significant role in the functional metabolism of ovarian cells. Prior research has established MI as a key molecular mediator in intercellular and intracellular communication, pivotal in signaling pathways associated with reproduction, hormonal regulation, and metabolism. Although high-level evidence supporting the use of inositol for the treatment of PCOS is currently lacking from an evidence-based medicine perspective, clinical trial outcomes suggest that inositol supplementation in PCOS patients can exert insulin-sensitizing effects akin to metformin (Greff et al., 2023). The regulatory effect of inositol on the gut microbiota remains understudied. Authoritative evidence from Cell Host & Microbe demonstrates that, in obese individuals at high risk of IR, shotgun metagenomic sequencing revealed a distinct microbiota structure characterized by a *Megamonas*-dominated, enterotype-like cluster. In-depth analysis indicated the presence of inositol-degrading genes in these microbes, leading to reduced inositol levels, increased intestinal fatty acid absorption, and consequently, the promotion of obesity (Wu et al., 2024). While the study subjects were not women with PCOS, it is anticipated that future research will elucidate the relationship between inositol and the gut microbiota in the context of PCOS as investigations into the role of the gut microbiota in PCOS progress.

## 6 Outstanding and future perspective

Despite changes in gut microbiota composition and function in PCOS, the research on PCOS microbiota appears observational due to its high heterogeneity, lacking specific alterations. IR is the most common complication of PCOS. Although dysbiosis has been understood to promote IR development, the mechanisms through which gut microbiota participate in IR occurrence in PCOS patients remain an area ripe for development, involving multifactorial and multi-stage contributions to IR-PCOS. Therefore, deciphering and annotating the PCOS gut microbiome and metabolome are essential paths forward to tackle reproductive disorders and global metabolic health challenges. Additionally, due to the majority of studies being cross-sectional, there is an urgent need for bioinformatics algorithms or longitudinal data predicting short-term or long-term dynamic changes in gut microbial communities. Lastly, despite preliminary insights into how gut microbiota participate in IR occurrence in PCOS patients in recent years, little focus has been placed on the clinical potential of gut microbiota to guide IR treatment. In the future, as research deepens into gut bacteriomics, metabolomics, and the interaction between microbial systems and the human body, modulating gut microbiota will inevitably emerge as a novel target for IR-PCOS.

## 7 Conclusion

In various studies linking dysbiosis to PCOS, gut microbiota imbalance has increasingly emerged as a significant characteristic of PCOS patients. This review summarizes the diverse microbial changes observed in different PCOS studies and underscores the critical role of both microbes themselves and their metabolites in the development of IR in PCOS patients. Finally, we emphasize the importance of regulating gut microbiota for treating IR-PCOS, highlighting the potential for future microbiota-based therapies to achieve personalized treatment for PCOS.
